# The Shape-Memory Effect of Hindered Phenol (AO-80)/Acrylic Rubber (ACM) Composites with Tunable Transition Temperature

**DOI:** 10.3390/ma11122461

**Published:** 2018-12-04

**Authors:** Shi-kai Hu, Si Chen, Xiu-ying Zhao, Ming-ming Guo, Li-qun Zhang

**Affiliations:** 1 Key Laboratory of Beijing City on Preparation and Processing of Novel Polymer Materials, Beijing University of Chemical Technology, Beijing 100029, China; 2017400073@mail.buct.edu.cn (S.-k.H.); aircs123@163.com (S.C.); guomm57@swu.edu.cn (M.-m.G.); zhanglq@mail.buct.edu.cn (L.-q.Z.); 2 SINOPEC Beijing Research Institute of Chemical Industry, Beijing 100013, China

**Keywords:** acrylic rubber, shape-memory polymer, hindered phenol, hydrogen bonding

## Abstract

To broaden the types and scope of use of shape-memory polymers (SMPs), we added the hindered phenol 3,9-bis[1,1-dimethyl-2-{b-(3-tert-butyl-4-hydroxy-5-methylphenyl)propionyloxy}ethyl]-2,4,8,10-tetraoxaspiro-[5,5]-undecane (AO-80), which comprises small organic molecules, to acrylic rubber (ACM) to form a series of AO-80/ACM rubber composites. The structural, thermal, mechanical property, and shape-memory properties of the AO-80/ACM rubber composites were investigated. We identified the formation of intra-molecular hydrogen bonding between –OH of AO-80 and the carbonyl groups and the ether groups of ACM molecules. The amount of AO-80 used can be adjusted to tailor the transition temperature. AO-80/ACM rubber composites showed excellent shape recovery and fixity. The approach for adjusting the transition temperature of AO-80/ACM rubber composites provides remarkable ideas for the design and preparation of new SMPs.

## 1. Introduction

**Figure 1:**
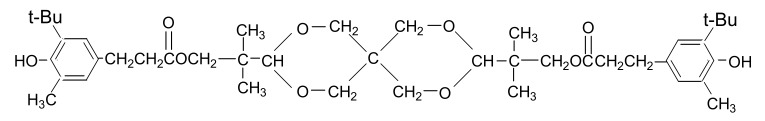
Chemical structure of hindered phenol 3,9-bis[1,1-dimethyl-2-{b-(3-tert-butyl-4-hydroxy-5-methylphenyl)propionyloxy}ethyl]-2,4,8,10-tetraoxaspiro-[5,5]-undecane (AO-80).

Shape-memory materials (SMMs) can change from one pre-determined shape to another in response to a certain stimulus [1,2]. Research on shape-memory polymers (SMPs) can be fundamental and applied. SMPs possess many advantages over their well-investigated metallic counterparts, shape-memory alloys; these advantages include excellent processability, light weight, and notable flexibility in terms of material design [3,4,5]; SMP applications include medical devices, actuators, sensors, artificial muscles, switches, smart textiles, and self-deployable structures [4,5,6,7]. SMPs can return into an original shape upon the application of stimuli, such as temperature [8,9,10], humidity [11,12], light [13,14,15,16], electricity [8,17,18,19,20], pH [15,21,22,23,24], and irradiation. This memory phenomenon is because a polymer network has reversible and fixed phases. The reversible phases can be shaped under certain conditions. Reversible phases use ionic bond [1,25], vitrification [25,26], reversible crystallization [27], hydrogen bond [28,29], or supramolecular interactions [30,31] to maintain this metastable shape until an activation energy is used to facilitate a return to the original shape. The fixed phases allow deformation but hold the relative location of the chains. Fixed phases include physical and covalent cross-links, such as crystalline or glassy domains in polymers, or supramolecular interactions [32]. For thermally induced SMPs, when the deformation of SMP is above its switch transition temperature (*T_trans_*) and then cooled below *T_trans_*, most internal stress can be stored in cross-linking structure; by heating the SMP above its *T_trans_*, the SMP recovers its original shape by releasing the internal stress [33,34]. When reheated above *T_trans_* without stress, the cross-linking phase assumes its permanent shape. *T_trans_* can either be the glass transition temperature (*T_g_*) or melting temperature (*T_m_*) of polymers. In general, the temperature province of *T_trans_* of current SMMs reaches above room temperature. However, in specific conditions, such as deep-sea and polar region explorations, *T_trans_* of SMMs should be lower than room temperature and can be adjusted and controlled by specific methods. A critical parameter for SMPs lies in its shape memory *T_trans_*. For an amorphous SMP polymer, it is important to develop new methods to tailor its *T_g_*, which corresponds to its shape memory *T_trans_*. Zhao et al. created a nano- or molecule-scale-hindered phenol and polar rubber compound. Their research indicated that *T_g_* of the developed material could be tailored by changing the kind and dosage of small organic molecule-hindered phenol [35,36]. This phenomenon was attributed to hydrogen bonding between hindered phenol 3,9-bis[1,1-dimethyl-2-{b-(3-tert-butyl-4-hydroxy-5-methylphenyl)propionyloxy}ethyl]-2,4,8,10-tetraoxaspiro-[5,5]-undecane (AO-80) and polar rubber. Such interactions will result in the molecular-level dispersion of AO-80 in CPE and rubber matrix and enhancement of intermolecular friction, which will further increase *T_g_*. It is well known that typical epoxy-based materials which have been applied extensively in coatings, adhesives, and matrix material for structural composites are rigid with relatively low failure strains. There are many references regarding shape-memory epoxy composites that all have good shape memory with a high shape fixity (*R_f_*) ratio and high shape recovery ratio (*R_r_*), but these composites all have a short elongation at break [37,38,39,40,41,42]. In this study, AO-80 had been studied to prepare AO-80/acrylic rubber (ACM) nanocomposites with high failure strains compared to shape-memory epoxy composites. The structure of AO-80 is shown in Figure 1. AO-80/ACM rubber nanocomposites possibly possess remarkable filler/matrix interfacial properties because the AO-80 molecule features numerous polar functional groups (hydroxyl and carbonyl) that can form strong intermolecular interactions with ACM. An elastomer will exhibit shape-memory functionality when the material can be stabilized in the deformed state in a temperature range that is relevant for particular applications. Similar to normal polymers, SMPs also possess 3D molecular network-like architectures. ACM can exhibit 3D network structures after crosslinking. These cross-linked structures ensure that the polymer can maintain a stable shape at the macroscopic level by enabling the original and recovered shapes. This system also features a *T_g_* below the room temperature, and temperature can be adjusted and controlled within a particular scope by incorporating small organic molecules to increase *T_g_* [35,36], which will broaden the kind and scope of use of SMPs. In this study, we designed a series of AO-80/ACM rubber composites with high failure strains, the *T_trans_* of which can be tailored by adding a dosage of small organic molecule-hindered phenol. No study or similar work has investigated the shape-memory effect of AO-80/ACM rubber composites, thereby broadening the list of SMPs with excellent shape-memory properties.

## 2. Materials and Methods

### 2.1. Materials

ACM (AR-801) was provided by Tohpe Corp (Sakai, Japan). AO-80 was obtained from Asahi Denka (Tokyo, Japan). Other ingredients and chemicals were obtained from China and were used as received.

### 2.2. Sample Preparations

AO-80/ACM rubber composites were obtained as follows: (1) After ACM was kneaded for 3 min, AO-80 (without previous treatment) was added into ACM. (2) After these mixtures were kneaded for 5 min, the AO-80/ACM mixtures were blended with compounding and crosslinking additives, including 5.0 phr of zinc oxide(CAS No:1314-13-2), 1.0 phr of stearic acid(CAS No: 57-11-4), 0.5 phr of potassium stearate(CAS No: 593-29-3), 4 phr of sodium stearate(CAS No: 822-16-2), and 0.5 phr of sulfur(CAS No: 7704-34-9). The mixtures were then kneaded for 10 min. The mixtures of AO-80/ACM were kept for at least 24 h. (3) Finally, the mixtures of AO-80/ACM were set at 180 °C and 15 MPa for 20 min and then naturally cooled down to prepare AO-80/ACM rubber composites.

### 2.3. Methods

The structure, shape-memory properties, and mechanical and thermal properties of AO-80/ACM rubber composites were systematically evaluated by differential scanning calorimetry (DSC), dynamic mechanical analysis (DMA), and Fourier-transform infrared (FT-IR) spectroscopy. The DSC curves were acquired from −60 °C to 150 °C at a rate of 10 °C/min with a STAR^e^ system calorimeter (Mettler–Toledo Co., Zurich, Switzerland). FT-IR spectra were acquired by using a Spectra-Tech ATR attachment to scan the samples.

The static mechanical properties of AO-80/ACM rubber composites were determined according to ASTM D638 by using a CMT4104 Electrical Tensile Tester (SANS Testing Machine Co., ShenZhen, China) at a rate of 500 mm/min at room temperature. The strip dimensions for testing were 20 mm in length, 6 mm in width, and 2 mm in thickness. Hardness was tested according to ASTM D2240-2015.

The shape-memory effect analysis of AO-80/ACM rubber composites was investigated on the DMA Q800 (TA Instruments, New Castle, DE, USA) using controlled-force mode with rectangular samples (6 mm in width and 2 mm in thickness). Prior to the investigation, the temperature was adjusted to an equilibration at *T_trans_* + 20 °C for 10 min. In step 1 (deformation), the sample was stretched to a designed value (*ε* = 55%, *ε* = 100%, *ε* = 130%) by ramping the force from a preload value of 0.005 N at a rate of 0.5 N/min. In step 2 (cooling), the specimen was cooled to fix the deformed sample under constant force at the rate of 3 °C/min to *T_trans_* − 20 °C. In step 3 (unloading and fixing), the force of the specimen was unloaded at a rate of 0.5 N/min to a preload value (0.005 N). Then, an equilibration at *T_trans_* − 20 °C for 10 min to ensure shape fixing was performed. In the final step (recovery), the specimen was reheated to *T_trans_* + 60 °C at the rate of 3 °C/min [37]. All experiments were carried out three times successively and the average results between second and third cycles are shown in the paper. From the curves, the shape recovery ratio (*R_r_*) and the shape fixity ratio (*R_f_*) for the shape-memory effect were computed as follows:

(1) $$\mathrm{Shape}\;\mathrm{recovery}:R_{r}\left(N\right)=\frac{\varepsilon_{m}-\varepsilon_{p}\left(N\right)}{\varepsilon_{m}-\varepsilon_{p}\left(N-1\right)},\;\times \;100\%$$

(2) $$\mathrm{Shape}\;\mathrm{fixity}:R_{f}\left(N\right)=\frac{\varepsilon_{u}\left(N\right)}{\varepsilon_{m}\left(N\right)}\;\times \;100\%$$

where $\varepsilon_{m},\varepsilon_{u}\;and\;\varepsilon_{p}$ are strains after the step of cooling, unloading, and recovery process, respectively. *N* refers to a consecutive number in a cyclic shape-memory measurement.

Dynamic mechanical properties were investigated on a DMA (Rheometric Scientific Co., Piscataway, NJ, USA). The strip dimensions for testing were 20 mm in length, 6 mm in width, and 2 mm in thickness. The curves of E′-T were acquired from −60 °C to 150 °C at a rate of 3 °C/min and with a frequency of 1 Hz at an amplitude of ε = 0.3%.

Shape recovery observations of the AO-80/ACM rubber composites were carried out in water. The composites were cut into rectangular strips with dimensions of 100.0 mm × 10.0 mm × 2.0 mm. The rectangular strips were fixed in a temporary shape at *T_high_* and then cooled down to *T_low_*. The rectangular strips in temporary shape were placed in a water bath at *T_high_* while recording images of shape recovery using a video camera at a rate of 20 frames/s. Among the aforementioned procedure/conditions, *T_high_* was equal to *T_trans_* + 20 °C, and *T_low_* was equal to *T_trans_* − 20 °C.

## 3. Results

### 3.1. T_g_ of AO-80/ACM Rubber Composites

**Figure 2:**
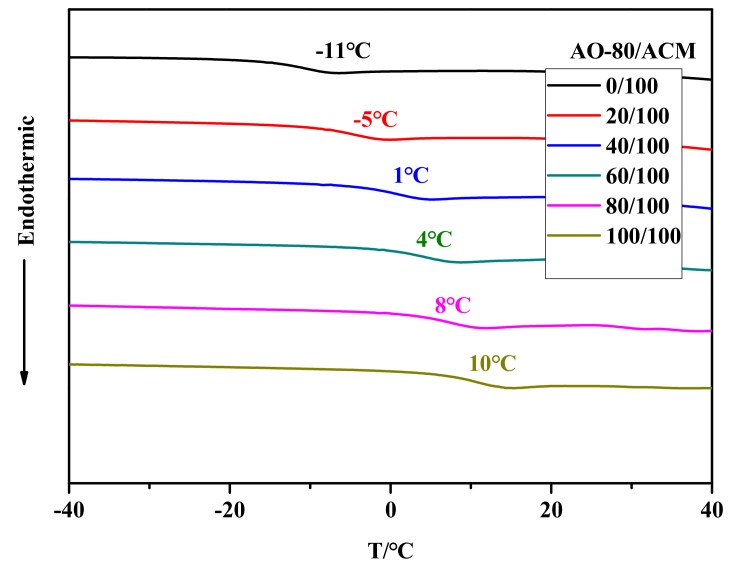
DSC curves of AO-80/acrylic rubber (ACM) rubber composites.

Figure 2 shows that the neat ACM featured a *T_g_* of approximately −11 °C. Compared with the neat ACM, AO-80/ACM composites showed a *T_g_* between those of neat ACM and quenched AO-80(40.9) [36]. *T_g_* of AO-80/ACM rubber composites shifted from −11 °C to 10 °C when the dosage of AO-80 was added from zero phr to one hundred phr. The DSC curves of the composites showed neither *T_g_* peak nor melting of AO-80 [36,43], which suggest that dispersion of AO-80 in ACM was at the molecular level by blending, and AO-80/ACM rubber composites were successfully prepared as expected. Strong intermolecular interactions were formed between AO-80 molecules and polar functional groups (ester and ether groups) of ACM. Hydrogen bonding between ACM and AO-80 are analyzed later. With both polar molecules, intermolecular interactions significantly hindered the slide of ACM chain and increased *T_g_* of ACM composites.

### 3.2. FT-IR of AO-80/ACM Rubber Composites

**Figure 3:**
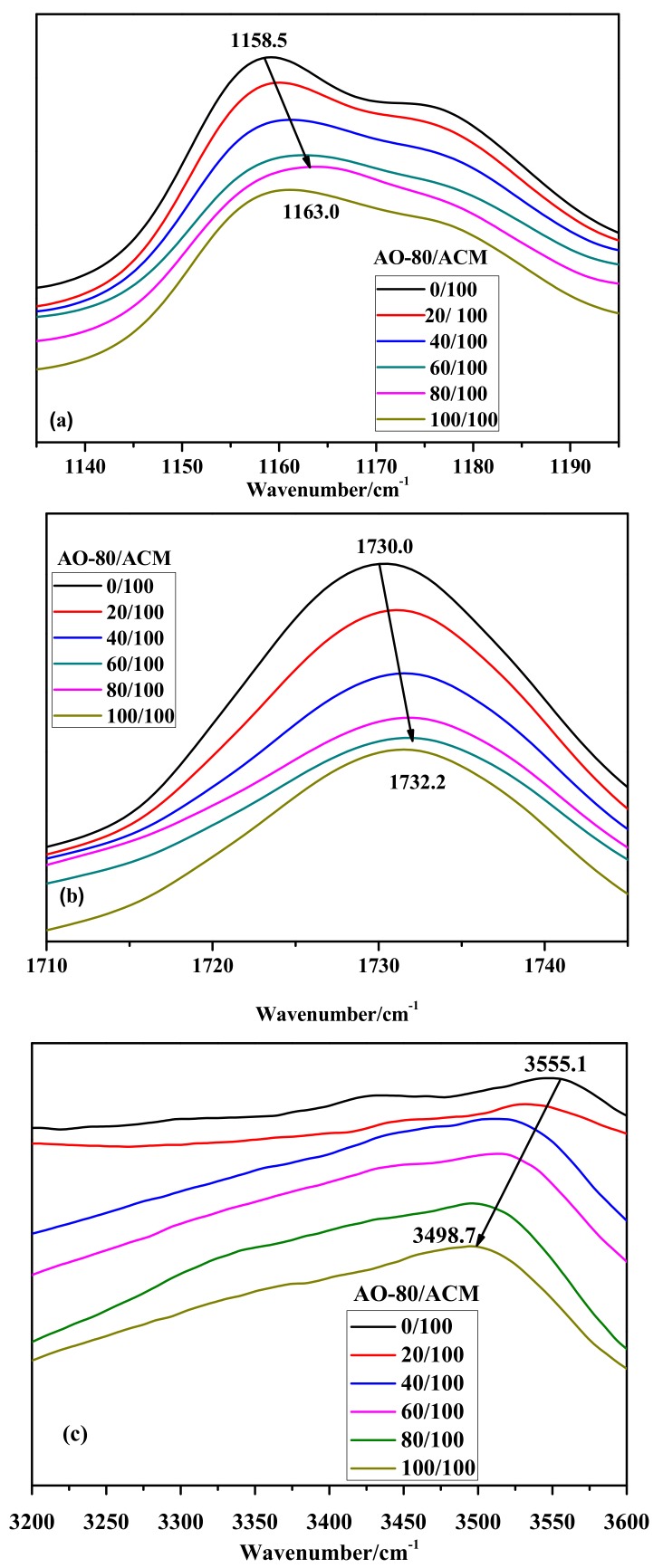
FT-IR spectra acquired at: (**a**) 1135 cm^−1^ to 1195 cm^−1^; (**b**) 1710 cm^−1^ to 1745 cm^−1^; and (**c**) 3200 cm^−1^ to 3600 cm^−1^ region for AO-80/ACM rubber composites.

**Figure 4:**
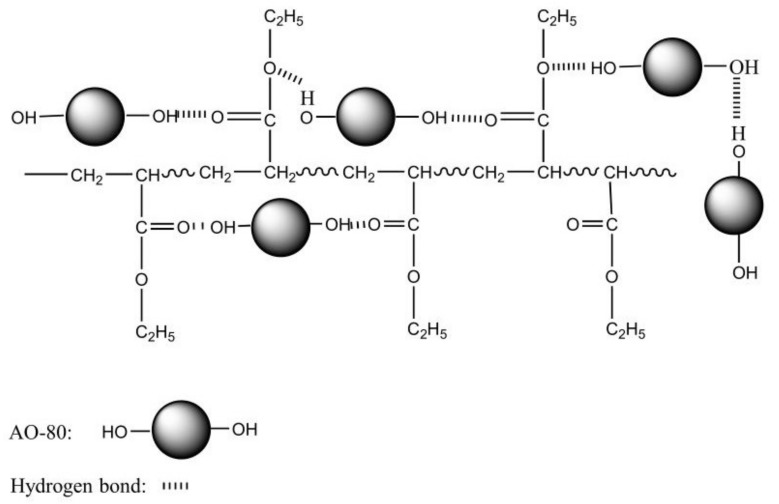
Possible hydrogen bond between AO-80 and ACM.

Interactions between different functional groups can be investigated through molecular dynamics simulation and FT-IR [44,45]. Figure 3 shows the FT-IR/ATR spectra of neat ACM and AO-80/ACM rubber composites. Figure 3a shows that the FT-IR/ATR spectra of all AO-80/ACM rubber composites indicate significantly wide peaks at 1135 cm^−1^ to 1195 cm^−1^, which were assigned to C-O-C bending vibration and symmetric and antisymmetric stretching vibrations. The peak position gradually shifted to a higher wave number from 1158.5 cm^−1^ to 1163 cm^−1^ when the dosage of AO-80 was added from zero phr to one hundred phr, determining that -O- of C-O-C can bond with-OH of AO-80. Figure 3b shows the composition dependence of FT-IR spectra for the –C=O stretching regions of AO-80/ACM rubber composites. As AO-80 content increased, the –C=O peak position shifted to a higher wave number from 1730.0 cm^−1^ to 1732.0 cm^−1^ when the dosage of AO-80 was added from zero phr to one hundred phr. Studies reported that hydrogen-bonded vibration will present a frequency shift [35,36]. Figure 3c shows the –OH stretching regions of AO-80/ACM rubber composites. The position of–OH peak shifted to a lower wave number from 3555.1 cm^−1^ to 3498.7 cm^−1^ when the dosage of AO-80 was added from zero phr to one hundred phr. The hydrogen bonding between carbonyl and ether groups of segments of ACM and -OH groups of AO-80 was observed. The total frequency shift as a measure of the strength of hydrogen bonding is generally accepted [46,47,48]. Thus, these results indicate that as the dosage of AO-80 increased, the strength of the hydrogen bonding among functional groups between ACM and AO-80 improved. The result corroborates that the *T_g_* of AO-80/ACM rubber composites increased with the dosage of AO-80, increasing because of hydrogen bonding. Figure 4 shows the possible hydrogen bonding of AO-80/ACM rubber composites.

### 3.3. Static Mechanical Properties of AO-80/ACM Rubber Composites

**Figure 5:**
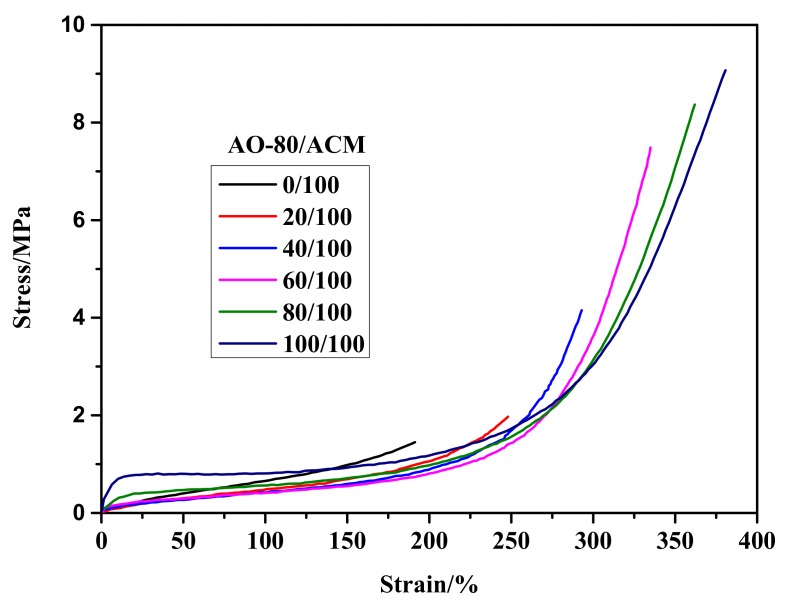
Stress-strain curves of ACM and AO-80/ACM rubber composites.

The results of the tensile testing of neat ACM and AO-80/ACM rubber composites are shown in Figure 5 and the acquired data is summarized in Table 1. The elongation and tensile strength at break of the neat ACM were 210% and 1.47 MPa, respectively. All of the AO-80/ACM rubber composites with a content of AO-80 above forty phr had much longer elongation and higher tensile strength at break than ACM. This was because AO-80 had a reinforcement effect when AO-80 was added over 40 phr and the strength of hydrogen bonding among functional groups between ACM and AO-80 was improved when the AO-80 content was added increasingly.

### 3.4. Shape-Memory Effect of AO-80/ACM Rubber Composite

**Figure 6:**
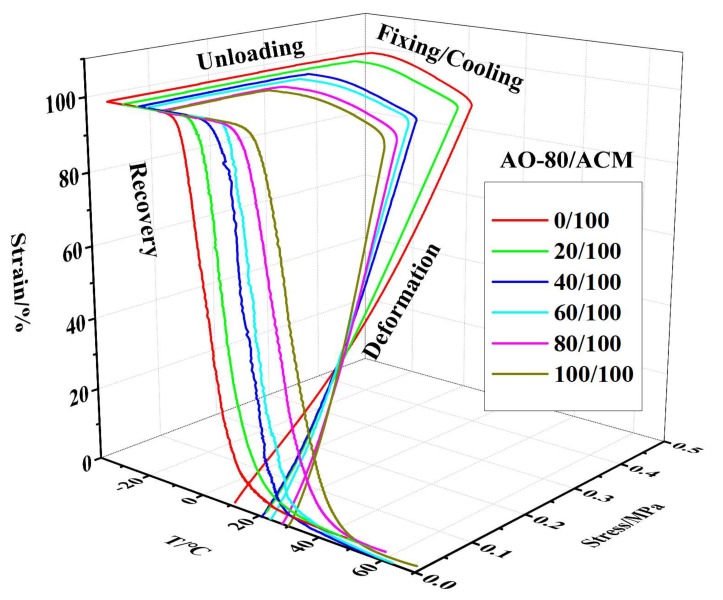
3D ε-T-σ curve of various compositions for AO-80/ACM rubber composites.

**Figure 7:**
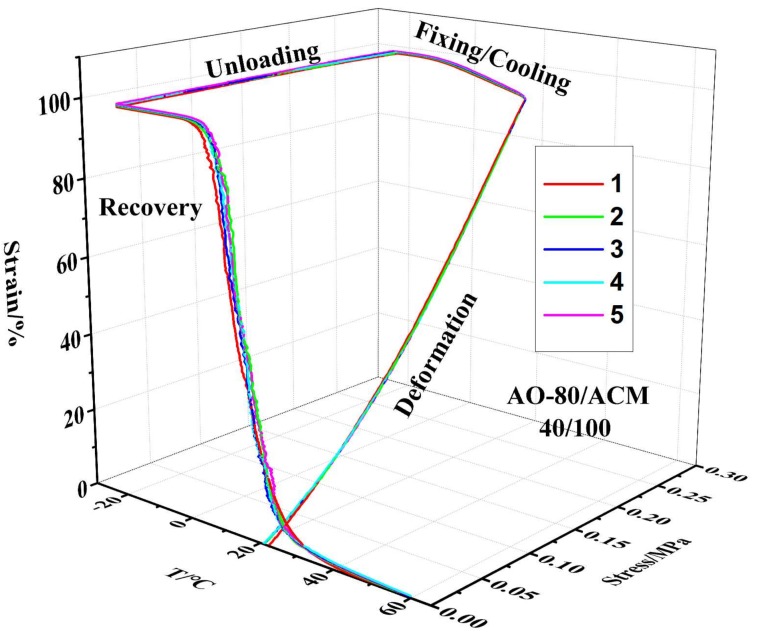
3D ε-T-σ curve of five cycles for AO-80/ACM (40/100) rubber composite.

**Figure 8:**
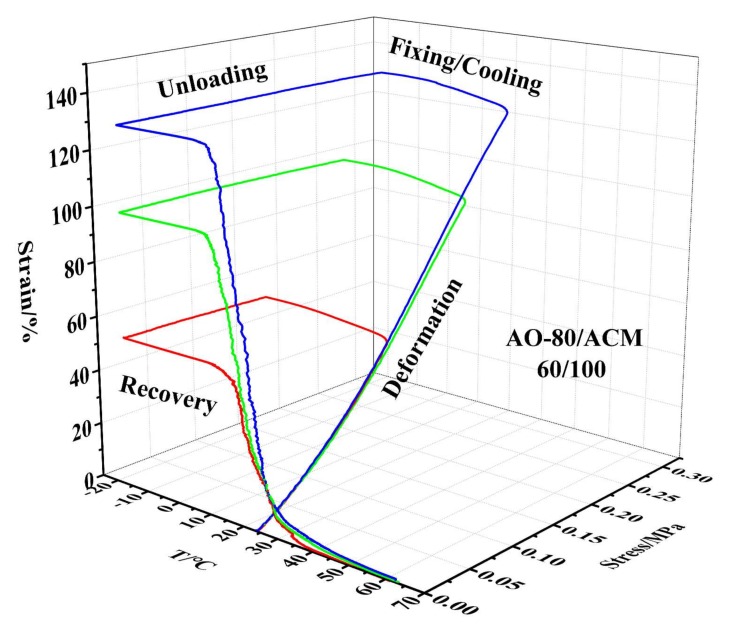
3D ε-T-σ curves of different strains (deformation) for AO-80/ACM (60/100) rubber composite.

**Figure 9:**
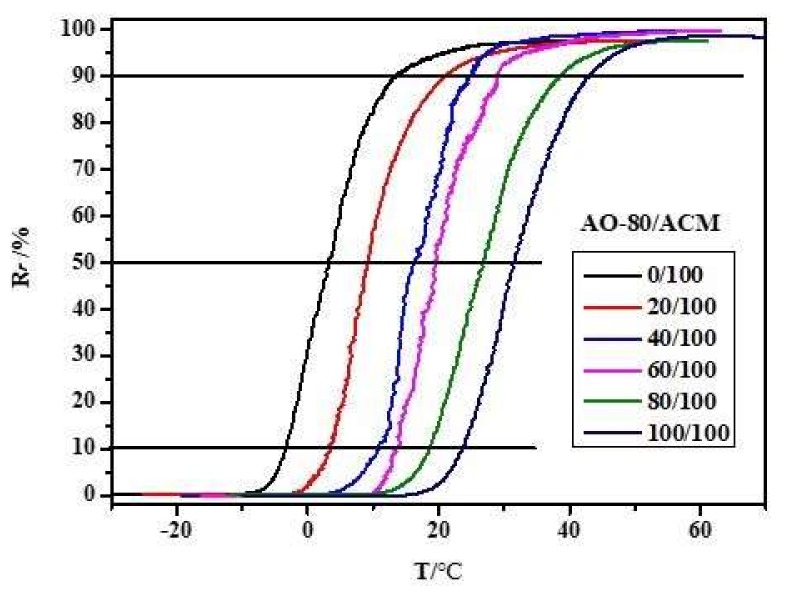
*R_r_*–T curves of AO-80/ACM rubber composites.

Figure 6 depicts the 3D ε-T-σ curves of various compositions for AO-80/ACM rubber composites. The results showed that the samples were generally further deformed because of loading during the cooling/fixing step after deformation, and the *T_g_* of AO-80/ACM rubber composites increased with an increasing dosage of AO-80; in other words, the *T_trans_* of AO-80/ACM rubber composites also increased with increasing AO-80. All samples exhibited excellent shape recovery, as shown in Figure 6. All the samples presented a high shape fixing ratio and recovery ratio when they were stretched to a given strain (100%). *R_r_* and *R_f_* were both above 99%. Figure 7 plots the 3D ε-T-σ curves of five cycles for AO-80/ACM (40/100) rubber composite. The 3D ε-T-σ curves of AO-80/ACM (40/100) rubber composites were similar with different cycles. Different cycles all showed high shape fixing and recovery rates. The results showed the repeatability of AO-80/ACM rubber composites as shape-memory materials were excellent. The excellent repeatability of AO-80/ACM rubber composites was due to good elasticity of samples. Figure 8 plots the 3D ε-T-σ curves of different strains (deformation) for AO-80/ACM (60/100) rubber composite. All the diagrams show high shape fixing and recovery ratio when the given strains were 55%, 100%, and 130%. *R_r_* reached above 99%, and *R_f_* was above 99%. The results show that the range of deformation for the AO-80/ACM rubber composites as shape-memory materials is broad, which is due to high elongation at break of AO-80/ACM rubber composites. Figure 9 displays the *R_r_*-T curves of AO-80/ACM rubber composites with various compositions. A significant portion of prestrain was recovered in all samples within the temperature range of *T*_10_–*T*_90_. With increasing AO-80, the recovery temperature, *T*_10_ (*R_r_* = 10%), *T*_50_ (*R_r_* = 50%), *T*_90_ (*R_r_* = 90%) increased, which was due to intermolecular interactions significantly hindering the slide of ACM chain and increasing the *T_g_* (*T_trans_*) of AO-80/ACM rubber composites. Figure 6, Figure 7, Figure 8 and Figure 9 show that AO-80/ACM rubber composites exhibit excellent shape-memory behavior.

The possible molecular mechanism of AO-80/ACM rubber composites is that AO-80/ACM rubber composites consist of molecular switches that are temperature-sensitive netpoints. The permanent shape in AO-80/ACM rubber composites was determined by netpoints that are cross-linked by the cross-linking agent. The temporary shape was fixed by the vitrification of AO-80/ACM rubber composites. Samples can be deformed to a temporary shape above *T_trans_* + 20 °C, and the shape can be fixed at *T_trans_* − 20 °C under stress. When heated above *T_trans_* + 60 °C without stress, the specimen recovered its original shape because of the netpoints.

**Figure 10:**
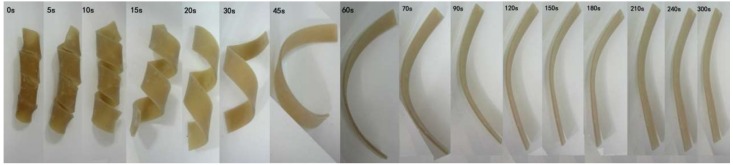
Shape recovery of AO-80/ACM rubber composites from a spiral-shaped temporary shape to stretched strip in water at 20 °C, which is higher than *T_g_*.

Figure 10 shows the shape-memory recovery of AO-80/ACM (100/100) rubber composite. After placing the components in water at 20 °C, which is higher than *T_g_*, they gradually recovered their original shape (Figure 10, t = 9 s–5 min). The results indicate that AO-80/ACM rubber composites exert shape-memory effects.

### 3.5. Dynamic Mechanical Properties of AO-80/ACM Rubber Composites

**Figure 11:**
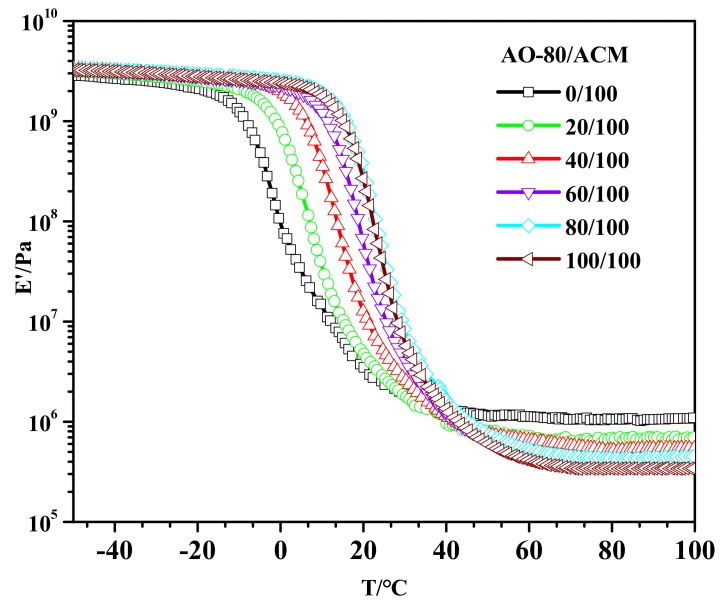
E′–T curves of AO-80/ACM rubber composites.

Dynamic mechanical properties of AO-80/ACM rubber composites are shown in Figure 11. All curves have only one transition, and the curves moved toward higher temperatures with an increasing dosage of AO-80. The E′ values of the AO-80/ACM rubber composites were similar in the glassy regions, whereas the E′ values in the rubbery regions decreased with an increasing dosage of AO-80. This was because the E′ values of AO-80 were similar to that of ACM matrix; therefore the E′ values of AO-80/ACM rubber composites were similar in the glassy state. When AO-80/ACM rubber composites were in the rubbery state, temperature was higher than the *T_g_* of AO-80 (40.9 C) [44], the AO-80 acted as a plasticizer after becoming soft, therefore the E′ values of AO-80/ACM rubber composites decreased. In AO-80/ACM rubber composites, all specimens showed a difference of approximately three orders of magnitude of AO-80/ACM rubber composites, which is responsible for the good recovery ratio and good shape fixity ratio for all specimens.

## 4. Conclusions

In this work, AO-80/ACM rubber composites were prepared. AO-80 has been successfully used to tailor *T_trans_* and *T_g_* of AO-80/ACM rubber composites became higher with the increment in AO-80. The formation of hydrogen bonding between carbonyl and ether groups of ACM molecules and the -OH of AO-80 is responsible for the increase in *T_g_*. Considering that *T_trans_* of ACM and AO-80/ACM rubber composites was related to *T_g_*, the *T_trans_* of AO-80/ACM rubber composites shifted from −11 °C to 10 °C when the dosage of AO-80 was added from zero phr to one hundred phr. In shape-memory experiments, the composites presented a shape-memory effect, and *T*_10_, *T*_50_, and *T*_90_ increased with *T_trans_*. Shape memory can be maintained at a wide deformation range and has good repeatability. All memory tests led to the conclusion that AO-80/ACM rubber composites feature excellent shape behavior. *R_f_* and *R_r_* of AO-80/ACM rubber composites were higher than 99% and 99%, respectively. The aforementioned approaches of tuning the transition temperature of developed composites can be potentially applied to other polymer systems.

## Figures and Tables

**Table 1:** Mechanical properties of AO-80/ACM rubber composites.

Properties	Loadings of AO-80/phr					
	0	20	40	60	80	100
Hardness (Shore A)	41 ± 0	48 ± 0	68 ± 0	78 ± 0	93 ± 0	95 ± 0
Tensile strength (MPa)	1.5 ± 0.2	1.9 ± 0.1	4.0 ± 0.2	7.7 ± 0.1	8.2 ± 0.1	9.2 ± 0.2
Elongation at break (%)	210 ± 9	248 ± 11	295 ± 12	336 ± 8	369 ± 8	377 ± 5
